# *Sestrin1, 2, and 3* are dispensable for female fertility in mice

**DOI:** 10.1186/s13048-024-01345-z

**Published:** 2024-02-01

**Authors:** Mengchen Wang, Wenhui Chen, Xinxin Zeng, Taojun Wang, Yingpu Sun, Qingling Yang

**Affiliations:** 1 Center for Reproductive Medicine, The First Affiliated Hospital of Zhengzhou University, 40, Daxue Road, Zhengzhou, 450052 Henan China; 2 Henan Key Laboratory of Reproduction and Genetics, The First Affiliated Hospital of Zhengzhou University, Zhengzhou, China; 3 Henan Provincial Obstetrical and Gynecological Disease (Reproductive Medicine) Clinical Research Center, The First Affiliated Hospital of Zhengzhou University, Zhengzhou, China; 4 Henan Engineering Laboratory of Preimplantation Genetic Diagnosis and Screening, The First Affiliated Hospital of Zhengzhou University, Zhengzhou, China

**Keywords:** Sestrin1, Sestrin2, Sestrin3, Female fertility, Ovarian aging

## Abstract

**Background:**

Sestrins have been implicated in regulating aging in various organs through multiple pathways. However, their roles in ovarian aging remain unrevealed.

**Methods:**

Female *Sestrin1*^*−/−*^, *Sestrin2*^*−/−*^, and *Sestrin3*^*−/−*^ mice were generated using the CRISPR-Cas9 system. Body weights, little sizes, ovarian weights, estrous cyclicity, and follicle number in female mice were observed. ELISA was utilized to measure serum anti-Müllerian hormone (AMH) levels. Real time PCR, western blot, immunofluorescence, and Masson trichrome staining were employed for assessment of aging-related change.

**Results:**

The deletion of *Sestrin 1, 2, or 3* had no discernible impact on body weights,or serum AMH levels in female mice at the age of 12 months. And there were no discernible differences in litter sizes or estrous cyclicity which were assessed at the age of 8 months. At the age of 12 months, no significant differences were observed in ovarian weights or follicle numbers among the knockout mice. Consistently, the extent of fibrosis within the ovaries remained comparable across all experimental groups at this age. Additionally, autophagy, apoptosis, DNA damage, and inflammation within the ovaries were also found to be comparable to those in wild-type mice of the same age.

**Conclusions:**

The loss of *Sestrin 1, 2, or 3* does not exert a noticeable influence on ovarian function during the aging process. *Sestrin1, 2, and 3* are not essential for female fertility in mice.

**Supplementary Information:**

The online version contains supplementary material available at 10.1186/s13048-024-01345-z.

## Introduction

The ovary is one of the earliest organs to age [1]. Ovarian aging encompasses the irreversible decline in reproductive capacity [2] and female ovarian reserve [3] alongside disturbances within the ovarian microenvironment [4], leading to increased ovarian fibrosis [5]. Female fertility experiences a decline due to ovarian aging [6], particularly after the age of 35 [7], as indicated by diminishing levels of serum anti-Müllerian hormone (AMH) [8], reduced follicle count [9] and altered menstrual cycle length [10]. While IVF/ICSI-ET is available for age-related infertility [11], it can be expensive and outcomes are not guaranteed [12]. With an aging population and more individuals delaying marriage and childbirth , comprehending the mechanisms behind ovarian aging becomes pivotal to identifying strategies for its delay, especially for women who intend to become pregnant later in life.

Sestrins, initially identified as p53 target genes that decline with aging [13], have been implicated in an array of cellular functions, including response to nutritional stress (liver) [14], metabolic regulation, and autophagy [15]. Sestrins are known to be potent antioxidants, primarily through the activation of the Nrf2-Keap1 pathway, promoting p62-dependent autophagic degradation [16]. Sestrins are also important negative regulators of rapamycin mTOR complex 1 (mTORC1) [17] aiding in the detoxification of harmful reactive oxygen species (ROS) [18] via activating adenosine monophosphate-activated protein kinase (AMPK) [19] which exerts an anti-aging influence [20]. The levels of ROS were observed to rise in cultured H2O2-treated RKO cells upon silencing *Sestrin2 (Sesn2)* [21], and to decrease in Akt1/2 DKO mouse embryonic fibroblasts (MEFs). *Sesn3* encoding two proteins with molecular weights of 44 kDa and 53 kDa [22], is a vital regulator of intracellular ROS down-stream via Akt and FoxOs pathway [23]. Ink4/Arf-tg/tg mice are protected against oxidative damage due to an increased expression of antioxidant genes (*Sesn1* and *Sesn2* in liver) mediated by p53 [24]. Sestrin1 (SESN1 ) also protected muscles against aging-induced atrophy [25]. Sestrin2 (SESN2), a 60 kDa protein [26] plays a protective role in the context of palmitate-induced lipotoxicity during pregnancy, regulating ER stress, inflammation, and apoptosis, and supports proper trophoblast invasion [27]. The Sestrin family of proteins have emerged as key regulators of aging processes. Multiple molecular markers, such as *p53, p16* and *p21*, change with aging [28]. Ovarian aging is a complex process that involves multiple molecular changes, including inflammation [29], autophagy [30], DNA damage [31], and apoptosis [32]. *Nlrp3* [7], *IL-1α* [33], and *TNF-α* [34] are vital inflammasome increasing with ovarian aging. The expression of p62, a component in autophagy, is increasing during ovarian aging [7]. DNA damage caused by ovarian aging can be revealed by accumulating γH2AX foci [31, 35].

Although, previous study shows that the inhibition of *Sestrin1* increases ROS generation and induced apoptosis in KGN cells (human granuloma-like tumor cells) [36], and SESN2 was reported participating in improving ovarian and follicular developmental in Chitooligosaccharide-zinc-treated premature ovarian failure mice [37]. The role of Sesntrin1, 2 and 3 in ovarian function was still largely unknown. Here, *Sesn1, 2*, and *3* knockout mice were generated by the CRISPR/Cas9 system. The ovarian functions were investigated in the knockout mice. Our data show that the deletion of any of these Sestrin family members did not exert any discernible influence on ovarian function.

## Materials and methods

### Animals

C57/BL6 wild-type mice were sourced from the Beijing Vital River Experimental Animals Centre in Beijing. *Sestrin1*^*−/−*^*(Sesn1*^*−/−*^*)*, *Sestrin2*^*−/−*^*(Sesn2*^*−/−*^*)*, and *Sestrin3*^*−/−*^*(Sesn3*^*−/−*^) were generated using the CRISPR/Cas9 system. DNA for genotyping was extracted from the mouse ears by a DNA extraction kit (Vazyme, Jiangsu China) and then amplified by primers with specific sequences (Supplementary Table 1). The amplified DNA was subjected to agarose gel electrophoresis and visualized under ultraviolet light. Homozygous knockout mice were obtained through the breeding of heterozygous knockout mice. Genotyping was performed on each knockout mouse before they were used in the experiments.

The mice were maintained under a 12-hour light/dark cycle at a temperature of 20–25 °C and provided with free access to food and water. All animal experiments were approved by the Ethics Committee of the First Affiliated Hospital of Zhengzhou University. The litter size of the knockout mice was determined by mating them with wild-type male mice, and the number of pups in each litter was counted immediately after birth through visual inspection.

### Immunohistochemistry

Immunohistochemistry (IHC) analysis was performed on ovary sections to identify the expression of Sestrin1, 2 and 3 (SESN1, 2 and 3). Ovarian tissue sections were deparaffinized, rehydrated, and subjected to antigen retrieval in 10 mM citrate buffer at 95 °C for 20 min. Primary antibodies, including Anti-Sestrin1 (Novus, NBP2-20316), Anti-Sestrin2 (Proteintech,10759-1-AP), and Anti-Sestrin3 (Proteintech,11431-2-AP), were applied at a 1:100 dilution and incubated overnight at 4 °C following the inhibition of endogenous peroxidase activity. HRP-conjugated secondary antibodies were utilized, and HRP activity was visualized with DAB. The negative control sections were performed with all steps identical to the procedure except for the addition of the primary antibodies.

### Estrous cycle

Vaginal cells were flushed with phosphate-buffered saline, smeared onto a glass slide. After fixation in 95% ethanol and staining with hematoxylin-eosin, slides were observed under a microscope over a period of 14 days. The estrus phase and the proportion of time spent in estrus phase in mice was evaluated as described earlier [38, 39].

### Serum anti-mullerian hormone (AMH) measurement

Serum samples from mice during the estrus phase were collected by extracting blood from medial canthus vein and subsequently stored at -80 °C until further use. The measurement of serum AMH levels was carried out using the Mouse AMH enzyme-linked immunosorbent assay (ELISA) Kit (Cusabio, Wuhan, China).

### Masson trichrome staining

Ovarian sections were prepared by embedding the ovaries from mice during the estrus phase in paraffin wax and cutting thin slices using a microtome. These sections were then mounted on microscope slides and subjected to Masson’s trichrome staining using a Masson’s trichrome stain kit (Solarbio, Beijing, China) following the manufacturer’s instructions without any modifications. This staining method facilitates the differentiation of collagen fibers, with collagen fibers appearing blue. Image analysis was conducted using ImageJ software to quantify the extent of collagen fiber deposition. Each section was carefully examined to measure both the area occupied by blue-stained collagen fibers and the total area of the section. The ratio of the blue-stained collagen fiber area to the total area was then calculated to assess the proportion of collagen fiber deposition in each section. This analysis yielded quantitative data regarding the extent of collagen fiber accumulation in the ovarian tissue sections.

### Real-time PCR assays

Total RNA was extracted from mouse ovarian tissues in the estrus phase utilizing TRIzol reagent, and complementary DNAs (cDNAs) were synthesized for subsequent real-time PCR analyses using a cDNA synthesis kit (Takara, Japan). Real-time PCR was performed using a Quant-studio 12 K Flex system from Applied Biosystems with a SYBR Green reaction mix (Qiagen, Germany). The expression levels of the target genes were calculated using the △△ CT method and were normalized to Gapdh. Primer sequences for the real-time PCR are provided in Supplementary Table 2.

### Western blotting

Proteins extracted from the ovarian tissue in the estrus phase were obtained using a protein extraction kit (Sangon Biotech, Shanghai, China) following the manufacturer’s instructions without any modifications. Following denaturation with reducing agents and SDS, these proteins were separated on a polyacrylamide gel, subsequently transferred to PVDF membranes (Bio-Rad Laboratories), and blocked with 5% skim milk (Servicebio) for 1 h at room temperature. The membrane was initially incubated with p62 primary antibody (1:1000, Cell Signaling Technology, #5114) overnight at four degrees Celsius, followed by incubation with a secondary antibody conjugated to a detection enzyme for 1–2 h. The protein was then detected by exposing the membrane to a chemiluminescence detection system (Bio-Rad, CA, USA). The bands’ intensity was quantified using Image J software, and the results were compared between samples.

### Sectioning ovaries and follicle counting

The ovaries from mice during the estrus phase were meticulously dissected to facilitate ovary sectioning and follicle counting. Following fixation, the ovarian tissues were embedded in paraffin wax. The 5 μm thick sections were then stained with H&E to visualize the follicles. Using a light microscope, the follicles in every fifth section were identified based on their distinctive morphology [40]. The total follicle number was counted by sum of follicle number from each section counted. They were further classified into different developmental stages, including primordial, primary, secondary, and antral follicles.

### Immunofluorescence

After being incubated in a 65 °C oven for one hour, paraffin sections were dewaxed and rehydrated using graded alcohol. Subsequently, the ovarian sections from mice during the estrus phase were blocked with ADB (PBS with 1% BSA and 0.1% Triton) at room temperature for 1 h before incubation with mouse anti-γH2AX primary antibody (Sigma, USA), which was diluted at 1:200. This incubation was carried out at four degrees Celsius overnight, followed by three washes with PBS for 15 min each. Next, the sections underwent an one-hour incubation with a fluorescent secondary antibody at 37 °C. Apoptosis in the ovarian tissue was detected using the TUNEL assay staining kit (Roche Diagnostics, IN, USA) [41]. Deparaffinized ovarian tissue sections were briefly rehydrated using graded alcohol after deparaffinization with xylene. TUNEL reagent was applied to the sections for one hour at 37 °C. Finally, the sections were counterstained with DAPI-containing anti-fade reagent, mounted, and examined under a Nikon microscope to calculate the positive rate. The nuclei were then identified using DAPI (Sigma, MO, USA). The quantification for Tunel and γH2AX positivity was performed by counting the number of positive cells per 100 granulosa cells within each antral follicle.

### Statistical analysis

All experiments presented in this study were conducted with a minimum of three replicates. The mean and standard error of the mean (SEM) were calculated using GraphPad Prism 10.0.0. Statistical analysis comparing wild-type (WT) and knockout mice was performed using an unpaired Student’s *t*-test. *P* < 0.05 was considered statistically significant. *P* values were represented as *P < 0.05, ns denotes *P* > 0.05.

## Results

### Female *Sestrin1*^*−/−*^, *Sestrin2*^*−/−*^, and *Sestrin3*^*−/−*^ mice do not exhibit fertility impairment

To explore the roles of the Sestrin protein family members within the ovaries, we examined the expression of these genes in WT mouse ovaries. Real time PCR results revealed that *Sesn1* and *Sesn2* exhibited a significant increase at 12 months of age, while *Sesn3* did not show any significant changes in expression within the ovaries of aging mice (Supplementary Fig. 1).

**Fig. 1:**
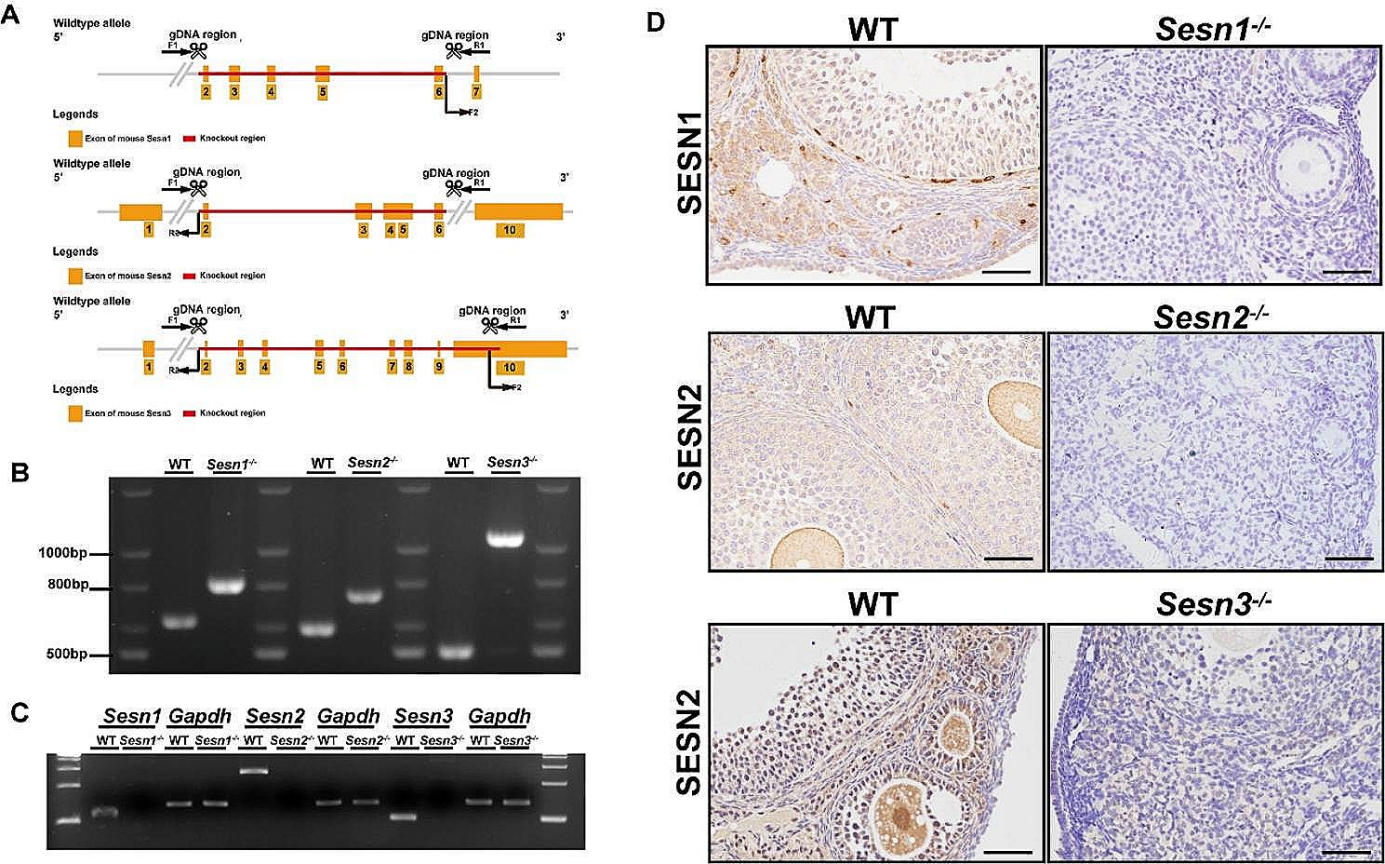
Construction of *Sesn1*^*−/−*^, *Sesn2*^*−/−*^, and *Sesn3*^*−/−*^ mice. (**A**) Limiting the expression of gene fragments for *Sestrin1*, *Sestrin2*, and *Sestrin3* using the CRISPR/Cas9 system. (**B**) Genotyping of wild-type (WT) and knockout mice. (**C**) Detection of S*esn1, Sesn2 and Sesn3* mRNA in *Sesn1*^*−/−*^, *Sesn2*^*−/−*^, and *Sesn3*^*−/−*^ and WT mice ovaries by Agarose gel electrophoresis of real time PCR. (**D**) Expression of SESN1, 2, and 3 in WT and knockout mice ovaries detected by IHC. Scale bar = 50 μm

To investigate the roles of the Sestrin protein family members within the ovaries, we employed the CRISPR-Cas9 system to generate whole-body knockout mice for each respective gene (Fig. 1A). Genotyping of different mouse strains within the Sestrin gene family was conducted using DNA gel electrophoresis (Fig. 1B). *Sesn1*^*−/−*^ mice exhibited a PCR product of 805 bp, while WT mice showed a band at 630 bp. *Sesn2*^*−/−*^ mice displayed a 738 bp PCR product, while WT mice had a 592 bp band. *Sesn3*^*−/−*^ mice were characterized by a 1067 bp PCR product, while WT mice showed a 499 bp band. The knockout efficiency was verified through qPCR using ovarian tissue samples (Fig. 1C), and the absence of Sestrin proteins in the ovaries of *Sestrin1*^*−/−*^(*Sesn1*^*−/−*^), *Sestrin2*^*−/−*^(*Sesn2*^*−/−*^), and *Sestrin3*^*−/−*^(*Sesn3*^*−/−*^) mice was confirmed by immunohistochemical analysis of paraffin-embedded ovarian sections (Fig. 1D). Additionally, Immunohistochemistry performed on ovarian sections from wild-type mice exhibited widespread expression of Sestrin family members within ovarian tissue (Fig. 1D).

**Fig. 2:**
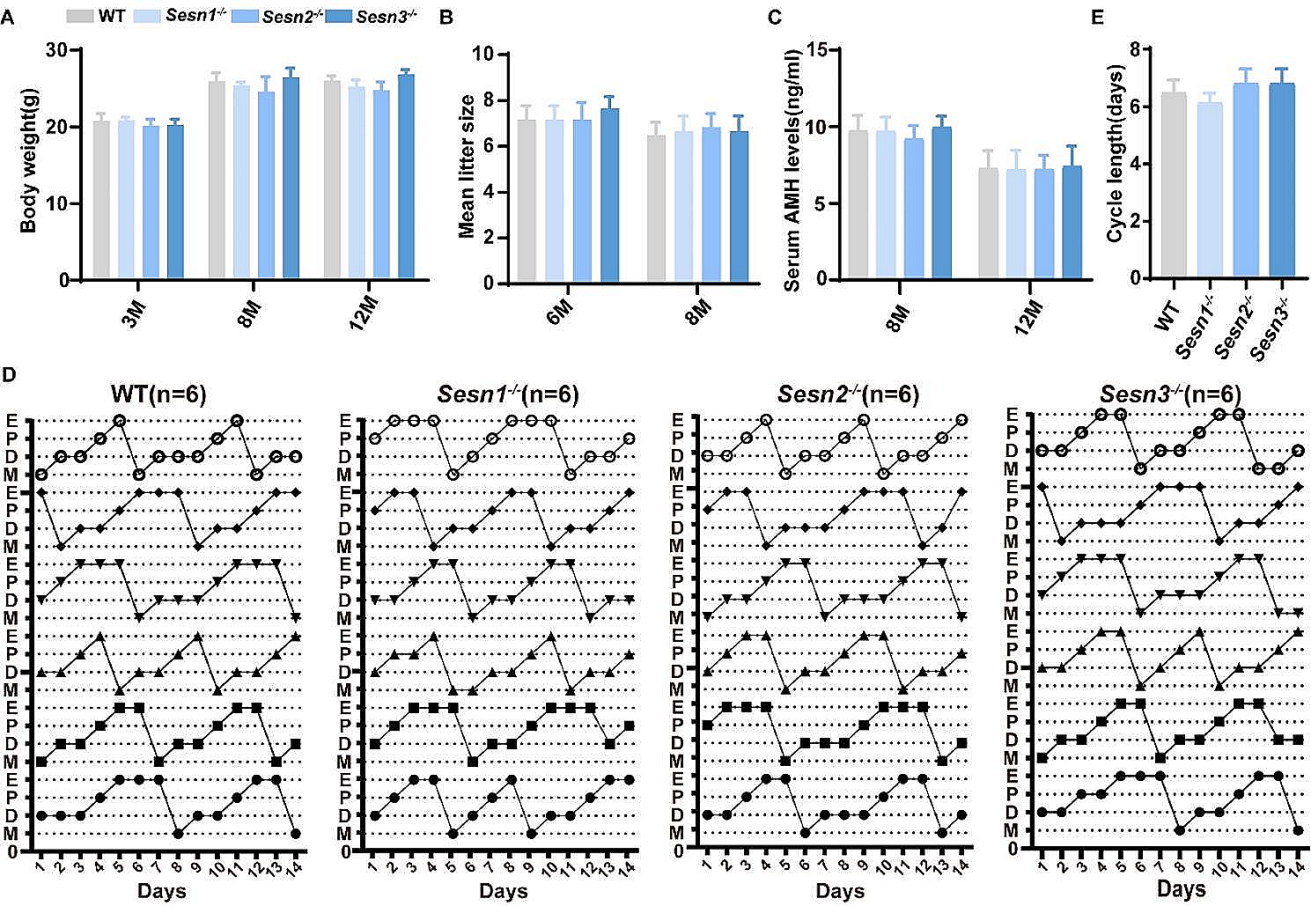
Assessment of fertility. (**A**) Body weights of 3-, 8-, and 12-month-old female mice (*n* = 6 mice for each group). (**B**) Mean litter sizes in 6- and 8-month WT, *Sesn1*^*−/−*^, *Sesn2*^*−/−*^, and *Sesn3*^*−/−*^ mice (*n* = 6 mice for each group). (**C**) Serum AMH levels of 8-month (8 M) and 12-month (12 M) WT, *Sesn1*^*−/−*^, *Sesn2*^*−/−*^, and *Sesn3*^*−/−*^ mice (*n* = 6 mice for each group). (**D**) Representative estrous cycles from 8 M WT, *Sesn1*^*−/−*^, *Sesn2*^*−/−*^, and *Sesn3*^*−/−*^ mice (*n* = 6 mice for each group). M, metestrus; D, diestrus; P, proestrus; E, estrus. (**E**) Length of estrous cycles (*n* = 6 mice for each group). Results are expressed as the mean ± SEM. Statistical differences between WT and knockout mice were measured by an unpaired Student’s *t* test

Subsequently, we proceeded to compare the fertility of both wild-type and knockout mice. Remarkably, at the age of 3, 8 and 12 months, neither the wild-type nor knockout mice exhibited notable differences in body weight (Fig. 2A), implying that the absence of any individual protein did not exert an influence on their weight. Further exploration involved a comparison of the average litter sizes between the 6-and 8-month-old wild-type and knockout mice and there is no discernible reduction in litter size among the knockout mice (Fig. 2B).

Serum anti-Müllerian hormone (AMH) levels serve as a dependable marker for assessing the quantitative aspect of ovarian reserve [8]. To assess this aspect, we obtained serum samples and subsequently quantified AMH levels. Our analysis of the collected data revealed no discernible impact on serum AMH levels in the 8 and 12 months knockout mice (Fig. 2C). Female C57BL/6 N mice naturally undergo four distinct estrous cycles: proestrus, estrus, metestrus, and diestrus [42]. The occurrence of irregular estrous cycles, encompassing prolonged or disrupted patterns, can serve as an indicator of reduced mouse fertility [43]. In our investigation, we meticulously examined vaginal smears from 8-month-old wild-type and knockout mice over a span of 14 days to comprehensively assess their estrous cycles. Notably, the duration of estrus in a complete cycle did no show significant differences between the wild-type and knockout mice (Fig. 2D). Importantly, the estrous cycle of knockout mice displayed no discernible aberrations, mirroring the patterns observed in the wild-type group (Fig. 2E). Given that mice are polytocous mammals [44], the evaluation of estrous cycles and litter sizes yielded no substantial differences between the wild-type and knockout mice. These findings collectively suggest that the Sestrin protein family may not assume a critical role in influencing female fertility in mice.

### Ovarian aging or diminished ovarian reserve was not observed in 12-month-old *Sestrin1*^*−/−*^, *Sestrin2*^*−/−*^, and *Sestrin3*^*−/−*^ mice

**Fig. 3:**
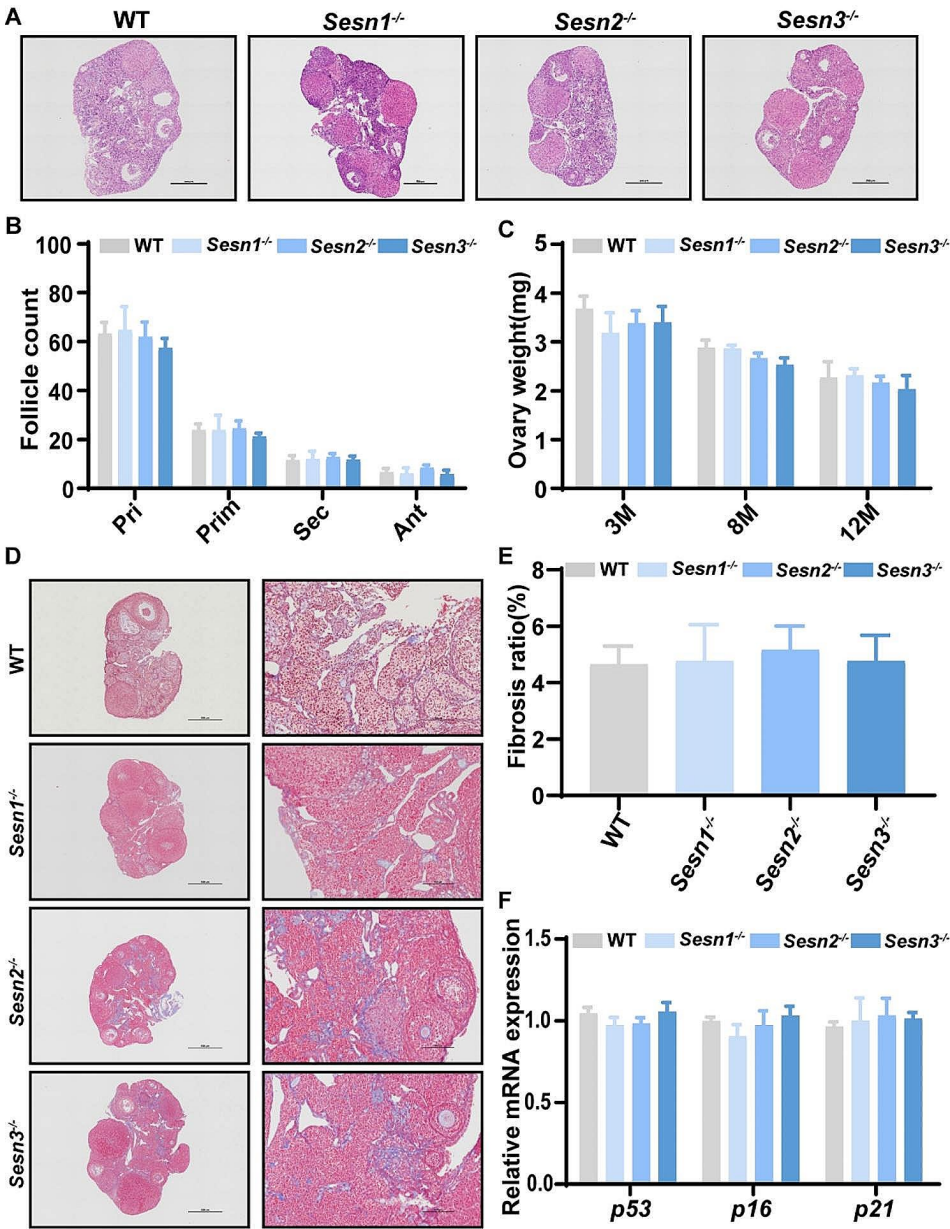
Comparison of ovarian reserve. (**A**) Representative micrographs of 12-month-old WT, *Sesn1*^*−/−*^, *Sesn2*^*−/−*^, and *Sesn3*^*−/−*^ mice ovarian sections. Scale bar = 500 μm. (**B**) Ovarian follicle count at developmental stages (primordial follicle (Pri), primary follicle (Prim), secondary follicle (Sec), and antral follicle (Ant) (*n* = 6 mice for each group). (**C**) Weights of ovaries in 3 M, 8 M, and 12 M WT, *Sesn1*^*−/−*^, *Sesn2*^*−/−*^, and *Sesn3*^*−/−*^ mice (*n* = 6 mice for each group). (**D**) Representative image of 12 months ovarian tissues with Masson’s trichrome staining. Scale bar = 500 μm. (**E**) The fibrosis area per ovarian section measured by ImageJ software. (**F**) The expression of *p16*, *p21*, and *p53* in 12 M ovaries (*n* = 6 mice for each group). *Gapdh* was used as a loading control. Results are expressed as the mean ± SEM. Statistical differences between WT and knockout mice were measured by an unpaired Student’s *t* test

As a crucial facet of our study, we investigated the role of the Sestrin protein family in both ovarian function and aging. Upon comparing wild-type and knockout mice at 12-month age, we intriguingly observed no substantial reduction in the number of follicles at any developmental stage (Fig. 3A, B). These compelling findings strongly implied that the absence of any Sestrin family member did not impede the progression of follicular development.

Furthermore, the ovarian weights exhibited parity between the wild-type and knockout mice at the age of 3, 8 and 12 months (Fig. 3C). We also meticulously assessed the 12 months ovarian fibrotic area. Ovarian fibrosis, characterized by excessive deposition of extracellular matrix, is recognized as a catalyst for ovarian dysfunction [45]. Employing Masson staining, we explored the extent of fibrosis within ovarian tissue, revealing no meaningful disparity in the proportional fibrotic content between the ovaries of wild-type and knockout mice (Fig. 3D, E).

The mRNA expression levels of age-related genes p53, p16, and p21 in 12-month ovarian, were assessed using quantitative PCR (qPCR) and were found to be consistent with the results obtained from Masson staining (Fig. 3F).

In summation, our findings indicate that the Sestrin protein family does not wield a substantial influence on ovarian reserve or the aging process within the ovaries.

### The loss of Sestrin1, Sestrin2, or Sestrin3 does not impact age-related autophagy, inflammation, or apoptosis in the ovaries

The aging process can induce cellular stress in the ovaries, resulting in disruptions to the ovarian microenvironment. Our comprehensive investigations, carried out on knockout mice to evaluate the impact of Sestrin protein family members on these processes within the ovaries, unveiled no significant differences when compared to wild-type mice.

**Fig. 4:**
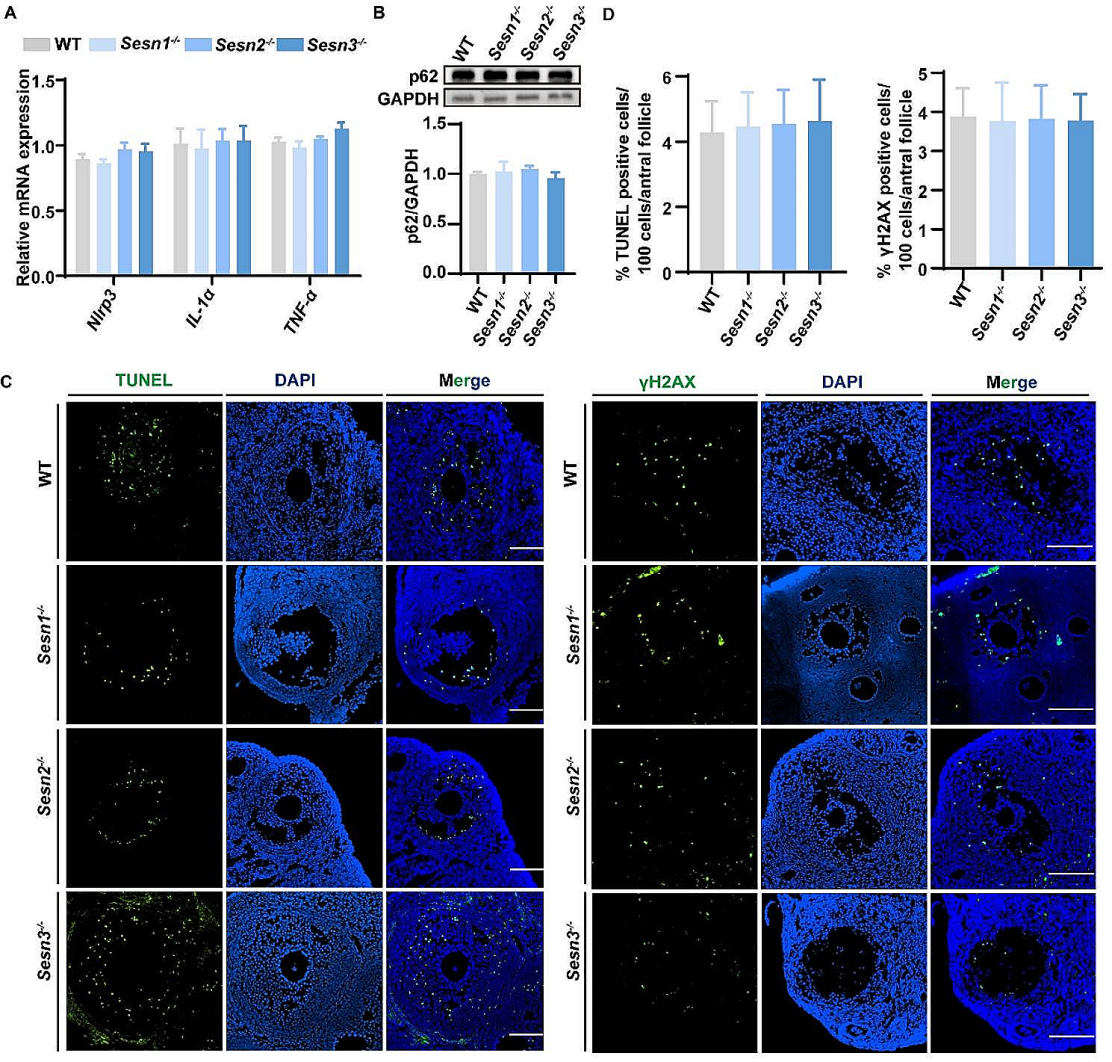
Analysis of aging processes in ovaries. (**A**) Relative mRNA levels of *Nlrp3*, *IL-1α*, and *TNF-α* (*n* = 6 mice for each group). (**B**) Western blot for p62 protein in ovarian. GAPDH was used as a loading control (*n* = 6 mice for each group). (**C**) Representative images of TUNEL and γH2AX staining in 12 M WT, *Sesn1*^*−/−*^, *Sesn2*^*−/−*^ and *Sesn3*^*−/−*^ mice. Scale bar = 100 μm. (**D**) Quantitative analysis of TUNEL and γH2AX positive cells (green) per 100 granulosa cells per antral follicle in WT, *Sesn1*^*−/−*^, *Sesn2*^*−/−*^ and *Sesn3*^*−/−*^ mice in 12 months of age (*n* = 6 mice for each group). Results are expressed as the mean ± SEM. Statistical differences between WT and knockout mice were measured by an unpaired Student’s *t* test

Specifically, the mRNA expression levels of inflammatory markers [46], including *Nlrp3* [7], *IL-1α* [33], and *TNF-α* [7, 34], demonstrated similarity across all four experimental groups (Fig. 4A). Consistently, in harmony with these findings, the results derived from Western blot analysis indicated that marker associated with autophagy, such as p62 [47], exhibited no significant deviation between the ovaries of 12 months wild-type and knockout mice (Fig. 4B). Moreover, by employing TUNEL and γH2AX immunofluorescence staining on ovarian tissue sections, we gained valuable insights into the presence and extent of granulosa cell apoptosis and DNA damage, respectively. The 12 months knockout mice did not manifest any substantial increase in granulosa cell apoptosis or DNA damage in comparison to their wild-type counterparts (Fig. 4C, D). These results indicate that even in the absence of a specific member, the cellular aging responses in the ovaries seem to be maintained at relatively normal levels. For further investigation into the interrelationships among Sestrin family members, real time PCR results revealed that the knockout of *Sestrin 1 or 2* only affected the expression of each other. Specifically, the deletion of *Sestrin 1* led to a decrease in the expression of *Sestrin 2*, while the knockout of *Sestrin 2* resulted in an increase in the expression of *Sestrin 1*. Notably, no significant effect on *Sestrin 3* was observed. In contrast, the knockout of *Sestrin 3* increased the expression levels of both *Sestrin 1 and 2* (Supplementary Fig. 2A-C). This suggests that the absence of one member can specifically influence the expression of other members, but it does not exert a substantial influence on cellular aging responses within the ovaries.

## Discussion

This study provides important insights into the involvement of Sestrins in female fertility and the cellular aging processes within the ovaries. However, the research results indicate that Sestrin1, Sestrin2, and Sestrin3 do not play a pivotal role in ovarian function, particularly concerning ovarian reserve and the acceleration of ovarian aging.

Sestrins, a family of stress-responsive proteins, have been extensively investigated for their roles in regulating aging and responding to various environmental stresses in different organs [48]. Sestrin1, 2, and 3 have been shown to regulate the accumulation of reactive oxygen species (ROS) by inhibiting the mechanistic target of rapamycin complex 1 (mTORC1) [20, 49]. Hyperactivation of mTORC1 can also suppress autophagy, which has the potential to reverse muscle atrophy [50]. Additionally, overexpression of Sestrin2 has been linked to decreased inflammation in osteoarthritis [51]. In this study, we employed the CRISPR-Cas9 system to create whole-body knockout mice for each Sestrin family member: Sestrin1, Sestrin2, and Sestrin3.

We found that the absence of Sestrins had no impact on ovarian aging and female fertility. Ovarian aging is a critical factor in female reproductive health, and comprehending the molecular mechanisms driving this process is of utmost significance, especially as more women delay childbirth. Age-related infertility often coincides with a decline in ovarian reserve [52]. Ovarian reserve refers to the quantity and quality of a woman’s remaining eggs (oocytes) within her ovaries [53]. Ovarian weights and follicle numbers can provide a quantitative measure of ovarian reserve, which showed no significant changes in knockout mice compared to their wild-type counterparts.

Ovarian aging is a complex process that involves multiple molecular markers providing insights into the cellular changes associated with aging. The expression levels of molecular markers associated with aging, such as p53, p16, and p21 [28], did not increase in the knockout group. Body weight can have a significant impact on fertility. Women who are significantly underweight or overweight may experience disruptions in their menstrual cycles and ovulation, which leads to irregular periods and reduced fertility [54]. Serum AMH levels [55] and litter sizes can also indicate fertility. Besides, regular menstrual cycles and consistent cycle lengths suggest healthy hormonal regulation and ovulatory function [10]. The knockout mice did not exhibit substantial differences in body weights, litter sizes, serum AMH levels, or estrous cyclicity.

Inflammation, autophagy and apoptosis, which are phenomena associated with aging in the body, also gradually manifest during ovarian aging [56]. In the ovaries, chronic or excessive inflammation contributes to ovarian aging [57], leading to the production of reactive oxygen species (ROS), which can damage ovarian cells and DNA. Autophagy leads to the accumulation of damaged cellular components [58], and impact the quality of eggs and the overall health of the ovaries [59]. Apoptosis is a natural process that helps regulate the number of follicles available for ovulation [60]. However, an imbalance in apoptosis, with either too much or too little cell death, can contribute to ovarian aging [61]. The level of inflammation, autophagy, and apoptosis detected in this report remained unchanged in knockout mice.

Previously, Sestrin1 [62], 2 [63] and 3 [64] all play roles in combating oxidative stress and regulating the redox balance within cells by inhibiting the mTOR signaling pathway and activating AMPK (AMP-activated protein kinase) [65]. Our data suggest that while the absence of one Sestrin family member does not impact the reproductive capacity and ovarian function in female mice, the loss of one member has been observed to specifically affect the expression of other members. Their potential interplay might be a contributing factor to why the knockout mice in this study did not exhibit a noticeable decline in fertility. It likely involves intricate signaling pathways and regulatory networks, necessitating further research to elucidate their detailed molecular mechanisms. The fact that the reproductive capacity and ovarian function of the knockout mice remained at normal levels in this study may suggest that the mice are still capable of coping with the effects of aging, perhaps due to their inherent self-regulatory mechanisms.

While Sestrins have been demonstrated to regulate aging in various organs, our findings suggest that the absence of Sestrin1, 2, or 3 does not significantly influence cellular aging responses in the ovaries. This research contributes to the expanding body of knowledge regarding the molecular mechanisms of ovarian aging and fertility, offering potential directions for further exploration in the quest to better comprehend and address age-related fertility challenges in women.

## Conclusions

The fertility, ovarian function, and ovarian aging processes in female *Sesn1*^*−/−*^, *Sesn2*^*−/−*^, and *Sesn3*^*−/−*^ is similar to the WT mice. This suggests that the function of Sestrin1, 2, and 3 in ovaries is not absolutely necessary.

### Electronic supplementary material

Below is the link to the electronic supplementary material.

Supplementary Material 1

## Data Availability

All data are available via the corresponding authors.
