# Supplementary material for: Sestrin1, 2, and 3 are dispensable for female fertility in mice
Source: J Ovarian Res. 2024 Feb 1;17:28. doi: 10.1186/s13048-024-01345-z (PMC10832176; doi:10.1186/s13048-024-01345-z)
Supplement: Supplementary file 1 — Supplementary Material 1 [file 13048_2024_1345_MOESM1_ESM.docx]

**Supplementary Materials for Sestrin1, 2, and 3 are dispensable for female fertility in mice**

Supplementary Table 1. Primer sequences for genomic DNA amplification

| Gene | | Forward | Reverse |
| --- | --- | --- | --- |
| *Sesn1* | Homozygotes | F1: GGCTTTCTGTTCCTGCTAATGTG | R1: CTGGGATAGACACTCAGGGGAT |
|  | Wildtype | F2: TTCGGGTAAGAACAGCCAGAATA | R1: CTGGGATAGACACTCAGGGGAT |
| *Sesn2* | Homozygotes | F1: TGGGGCTTGACCACTCTTTTCC | R1: ATACCTGGGCACGGAAGGTTG |
|  | Wildtype | F1: TGGGGCTTGACCACTCTTTTCC | R2: TAAAGCCAAGAGATCAGGCCAGC |
| *Sesn3* | Homozygotes | F1: CCTGAGGGAGGATGTGATGACGC | R1: CAGATAACCATGTGGGAAATGGGA |
|  | Wildtype | F2: GGCGAGTACTACTTTACGTTGTTA | R1: CAGATAACCATGTGGGAAATGGGA |

Supplementary Table 2. Primer sequences for real time PCR

| Target Gene | Forward | Reverse |
| --- | --- | --- |
| *Sesn1* | GGACGAGGAACTTGGAATCA | ATGCATCTGTGCGTCTTCAC |
| *Sesn2* | GAGTGCCATTCCGAGATCAAG | TAGTCCGGGTGTAGACCCATC |
| *Sesn3* | GGGCAGCAACTTTGGGATTG | CCATGAGCGCATCGCAATC |
| *p53* | GCGTAAACGCTTCGAGATGTT | TTTTTATGGCGGGAAGTAGACTG |
| *p16* | CGCAGGTTCTTGGTCACTGT | TGTTCACGAAAGCCAGAGCG |
| *p21* | CCTGGTGATGTCCGACCTG | CCATGAGCGCATCGCAATC |
| *Nlrp3* | ATTACCCGCCCGAGAAAGG | TCGCAGCAAAGATCCACACAG |
| *TNF-α* | GACGTGGAACTGGCAGAAGAG | TTGGTGGTTTGTGAGTGTGAG |
| *IL-1α* | CGAAGACTACAGTTCTGCCATT | GACGTTTCAGAGGTTCTCAGAG |
| *Gapdh* | CCTCGTCCCGTAGACAAAATG | TGAGGTCAATGAAGGGGTCGT |


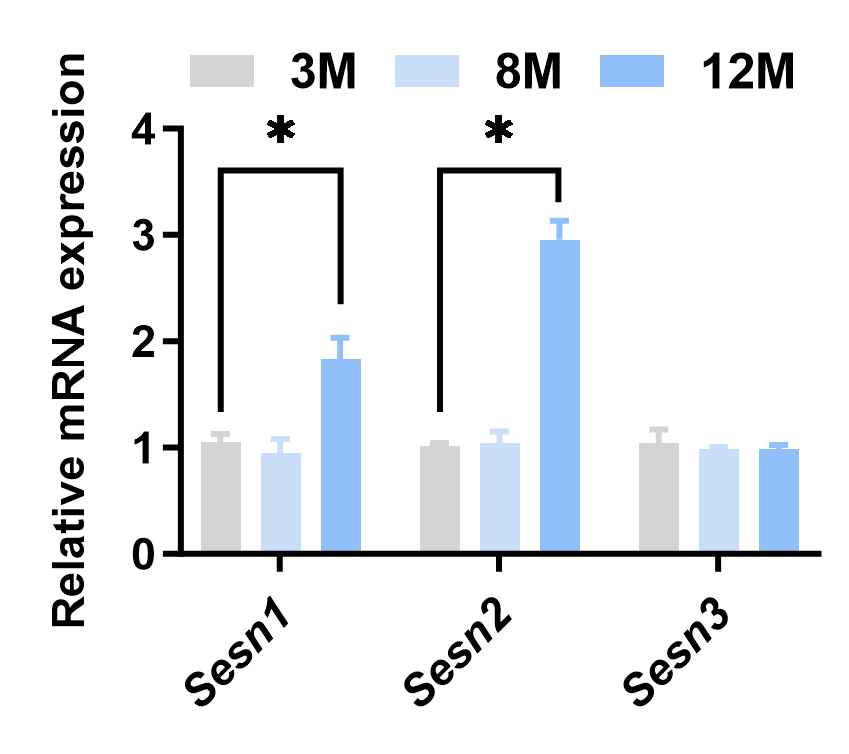


Supplementary Figure 1. The mRNA expression of S*estrin1, 2, and 3* in wild-type (WT) mouse ovaries with aging. The mRNA expression levels of *Sestrin1, 2, and 3* were assessed in the ovaries of WT mice at 3-, 8- and 12-month age through real time PCR (n=6 mice for each group). *Gapdh* was used as a loading control. Data were presented as mean ± SEM. *P* values were represented as **P* < 0.05.


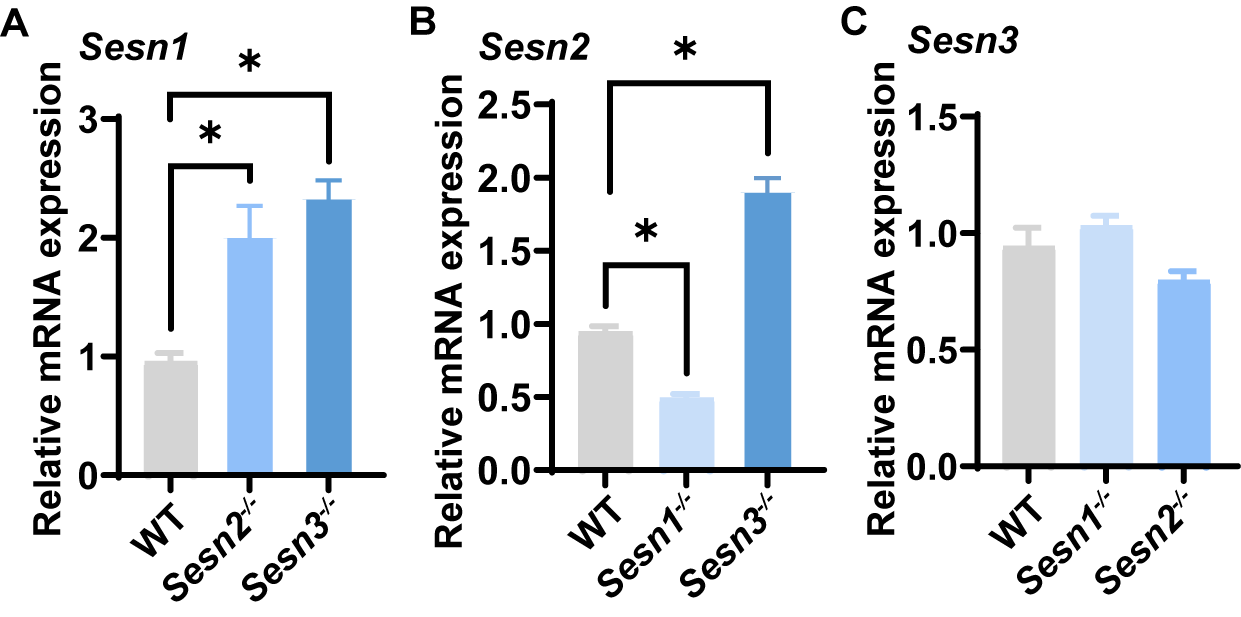


Supplementary Figure 2. The mRNA expression *Sestrin1, 2, and 3* in WT and knockout mouse ovaries.

(A) The mRNA expression levels of *Sesn1* were assessed in the ovaries of WT, *Sesn2^-/-^*, and *Sesn3^-/-^* mice at 12-month age (n=6 mice for each group). (B) The mRNA expression levels of *Sesn2* were assessed in the ovaries of WT, *Sesn1^-/-^*, and *Sesn3^-/-^* mice at 12-month age (n=6 mice for each group). (C) The mRNA expression levels of *Sesn3* were assessed in the ovaries of WT, *Sesn2^-/-^*, and *Sesn3^-/-^* mice at 12-month age (n=6 mice for each group). All these analyses were performed through real time PCR. *Gapdh* was used as a loading control. Data were presented as mean ± SEM. *P* values were represented as **P* < 0.05.
